# Balancing Accuracy and Computational Efficiency: A Faster R-CNN with Foreground-Background Segmentation-Based Spatial Attention Mechanism for Wild Plant Recognition

**DOI:** 10.3390/plants14162533

**Published:** 2025-08-14

**Authors:** Zexuan Cui, Zhibo Chen, Xiaohui Cui

**Affiliations:** 1 College of Information Science and Technology, Beijing Forestry University, Beijing 100083, China; zxtsui@bjfu.edu.cn (Z.C.); zhibo@bjfu.edu.cn (Z.C.); 2 Engineering Research Center for Forestry-Oriented Intelligent Information Processing of National Forestry and Grassland Administration, Beijing 100083, China; 3 Hebei Key Laboratory of Smart National Park, Beijing Forestry University, Beijing 100083, China

**Keywords:** wild plants recognition, lightweight bottleneck, spatial attention mechanism, unsharp masking, Faster R-CNN

## Abstract

Computer vision recognition technology, due to its non-invasive and convenient nature, can effectively avoid damage to fragile wild plants during recognition. However, balancing model complexity, recognition accuracy, and data processing difficulty on resource-constrained hardware is a critical issue that needs to be addressed. To tackle these challenges, we propose an improved lightweight Faster R-CNN architecture named ULS-FRCN. This architecture includes three key improvements: a Light Bottleneck module based on depthwise separable convolution to reduce model complexity; a Split SAM lightweight spatial attention mechanism to improve recognition accuracy without increasing model complexity; and unsharp masking preprocessing to enhance model performance while reducing data processing difficulty and training costs. We validated the effectiveness of ULS-FRCN using five representative wild plants from the PlantCLEF 2015 dataset. Ablation experiments and multi-dataset generalization tests show that ULS-FRCN significantly outperforms the baseline model in terms of mAP, mean F1 score, and mean recall, with improvements of 12.77%, 0.01, and 9.07%, respectively. Compared to the original Faster R-CNN, our lightweight design and attention mechanism reduce training parameters, improve inference speed, and enhance computational efficiency. This approach is suitable for deployment on resource-constrained forestry devices, enabling efficient plant identification and management without the need for high-performance servers.

## 1. Introduction

With the rapid development of computer vision technology, object detection and image classification techniques have been widely applied in fields such as forestry resource surveys, ecological monitoring, and biodiversity research [1,2,3]. Among these, wild plant recognition, as a key technology, directly impacts the effectiveness of forestry resource management, ecological conservation, and biodiversity studies through its accuracy and efficiency in detection and classification [4,5,6,7]. Current computer vision-based wild plant recognition methods can be broadly categorized into two types: traditional digital image processing methods [8] and machine learning-based approaches [9,10].

In the field of traditional image processing, researchers often employ plant phenotyping analysis, local feature fusion enhancement, and edge detection algorithms to extract single features for recognition. For example, Fansyuri et al. [11] proposed a flower recognition method combining color and shape features, using K-Means clustering for image segmentation and extracting HSV and YCbCr color features along with geometric features, achieving an accuracy of 71.1%. However, this method’s performance under uniform backgrounds struggles to generalize to complex natural environments. Kusnandar et al. [12] optimized HSV feature enhancement using the Heaviside step function, demonstrating advantages in color selection and processing speed for drone imagery. For plant texture and shape recognition, Seeland et al. [13] systematically evaluated various local feature representation methods and found that the Hessian-based SURF detector combined with Fisher Vector encoding yielded the best classification performance. Xu et al. [14] proposed a sequential morphological edge detection method that effectively extracts leaf edge features under natural lighting conditions while exhibiting strong noise resistance. Nevertheless, traditional methods generally rely on consistent image backgrounds and are susceptible to lighting variations and background interference in complex natural environments [15].

In recent years, deep learning methods have demonstrated significant advantages in wild plant detection due to their powerful feature extraction and generalization capabilities. Dyrmann et al. [16] compared the performance of ResNet50V2, MobileNetV2, and YOLOv3 under different parameters, finding that ResNet50V2 and MobileNetV2 achieved mean average precision (mAP) values of 58.5% and 72.3%, respectively, with large input sizes. James et al. [17] employed U-Net for processing drone imagery, achieving 96% accuracy through brightness enhancement and loss function optimization. Bhuyan et al. [18] introduced the CBAM attention mechanism into Res4Net, improving the F1 score for tea leaf disease detection to 98.37%. Yi et al. [19] enhanced InceptionV3 with a dual-path architecture and incorporated a center attention mechanism, achieving 77.1% accuracy in tree species recognition. To address multi-scale recognition, Boudra et al. [20] improved multi-scale local binary patterns, demonstrating excellent performance in bark image retrieval. Tian et al. [21] optimized U-Net encoding efficiency, achieving an F1-score of 95.9% in apple flower recognition. Although existing studies have made significant progress in detection accuracy, their high computational costs and training times limit their application in mobile and edge computing scenarios. Particularly in fully lightweight solutions capable of recognizing different plant parts in wild environments, current research remains insufficient.

To address these challenges, this paper proposes an improved algorithm based on Faster R-CNN, which features several novel contributions that enhance the performance and efficiency of wild plant recognition in resource-constrained environments. The key contributions are as follows:

1. Lightweight Feature Extraction Module: A bottleneck structure constructed using depthwise separable convolution significantly improves inference efficiency, addressing the computational limitations of forestry equipment.
2. Foreground-Background Segmentation-Based Spatial Attention Mechanism (Split SAM): This mechanism enhances focus on target regions and improves feature extraction by segmenting foreground and background, mitigating interference from complex environments.
3. Unsharp Masking Preprocessing: By enhancing edge features, this technique synergizes with the Split SAM attention mechanism, effectively balancing computational cost and detection performance on low-resource devices.

## 2. Materials and Methods

### 2.1. Image Dataset

#### 2.1.1. Dataset Labeling

**Figure 1:**
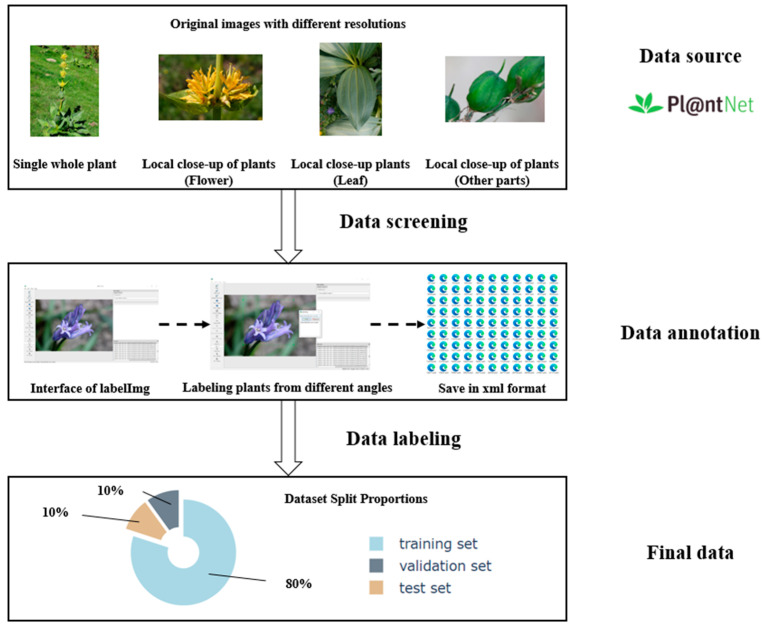
Data annotation process.

In the field of computer vision detection and recognition, data annotation is a crucial part of building and training models. It gives models clear learning goals and supervision info, which is key to making models more accurate and able to work well with different kinds of data. When data is annotated precisely, models can pick up on the important features and patterns. This helps them make good predictions, even with new data they haven’t seen before. Also, having high-quality annotated data is essential for putting together good datasets and for knowing how to make models better and more efficient. So, this section will lay out our approach to data annotation in detail, with the basic steps shown in Figure 1.

First, we selected a benchmark dataset—the PlantCLEF 2015 dataset [22], which consists of 113,205 images originating from 1794 observations, covering 1000 species of trees, shrubs, and ferns native to Western Europe. Each image falls into one of seven view types (entire plant, fruit, leaf, flower, stem, branch, and leaf scan) and is associated with a plant observation identifier to link it with other images of the same individual plant (observed by the same person on the same day). In this study, we filtered and selected field photographs and close-up images of five specific plant species, namely *Acanthus mollis* (Bear’s Breeches), *Cirsium eriophorum* (Woolly Thistle), *Gentiana lutea* (Yellow Gentian), *Hyacinthoides non-scripta* (Bluebell), and *Urospermum dalechampii* (False Dandelion), and used this filtered training set as the dataset for our experiments.

An important reason for choosing the PlantCLEF 2015 dataset is its socially contributed nature, which makes it close to real-world identification scenarios: (1) images of the same species come from plants growing in different regions; (2) images are taken by different users, who may not have followed the same image capture protocols; (3) images were taken at different times of the year.

**Figure 2:**
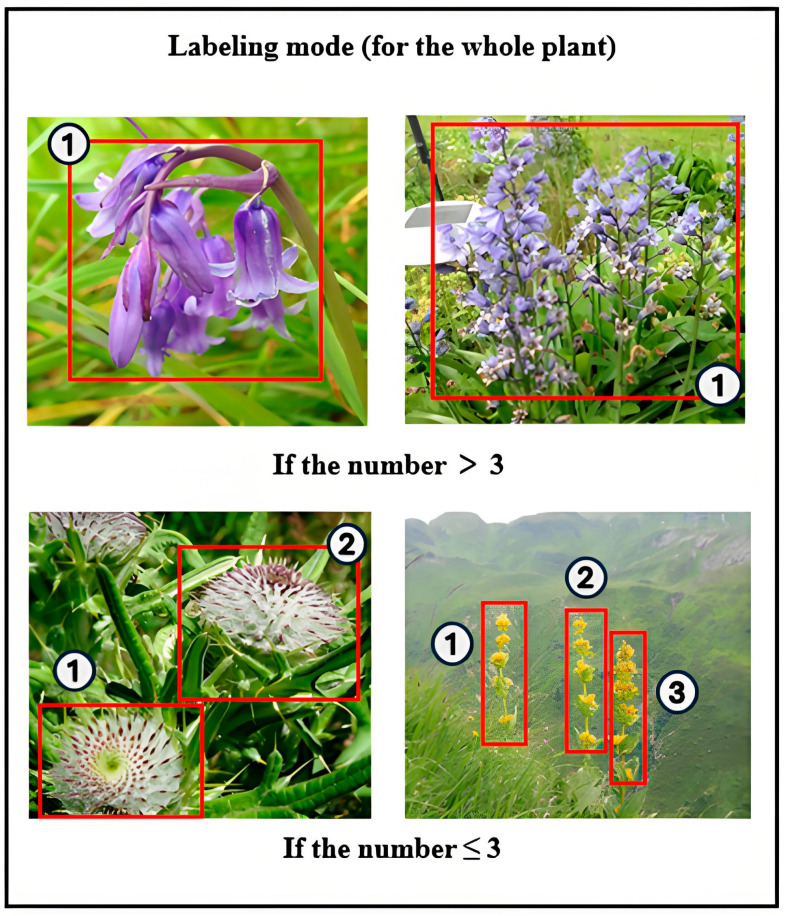
Data annotation strategy.

Secondly, we manually annotated the images using the LabelImg software (v1.8.6). Considering the complexity of the natural environment and the fact that individuals of the same plant species often grow in clusters, we adopted the following annotation strategy. Given the varying number of plant individuals or target local features (such as flowers) in the images, different annotation methods are applied. If the number exceeds three, range-based annotation is used; if it is three or fewer, each is annotated separately. Examples of this annotation strategy can be seen in Figure 2.

The annotation of the five different plant species was carried out using their Latin names as labels, and the label files were saved in the .xml format. Each annotation file contains image metadata, including bounding box information, image name, annotation label, and coordinate details. Ultimately, we organized the label files and the images stored in .jpg format according to the Faster R-CNN format, ultimately creating a dataset that comprises 531 images.

In addition, a data augmentation strategy was applied to the plant images during training, using the unsharp masking technique, as detailed in Section 2.1.2. It is important to note that the data augmentation did not alter the image format within the dataset, since these operations were performed online. Specifically, the augmentation was conducted in real time during training without changing the original storage format of the images (still in .jpg format). This approach allowed the model to encounter more diverse visual features during training, thereby improving its generalization ability and robustness in complex field environments.

Third, given the small scale of the dataset, a stratified sampling strategy was employed to divide the field plant dataset into training (429 images), validation (48 images), and test (54 images) sets in a ratio of 8:1:1. This split ensures a sufficient number of training samples while allowing hyperparameter tuning through the validation set and ultimately evaluating the model’s generalization performance on an independent test set [23]. As indicated in Table 1, each subset maintains the original class distribution of the dataset, which helps to avoid evaluation bias caused by data partitioning.

To further quantify and validate the unstructured and heterogeneous characteristics of the dataset, we conducted statistical and visual analyses of the image distribution from two dimensions: resolution and brightness. Overall, the dataset’s variety and consistent brightness levels make it a good representation of complex real-world situations, serving as a useful benchmark for testing how well models can handle different scenarios.

**Figure 3:**
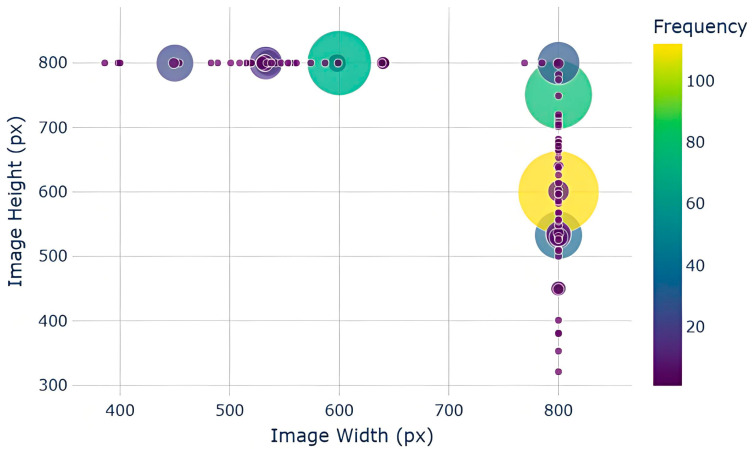
Image size bubble chart.

Specifically, we visualized the distribution of image dimensions using a bubble chart, with the x-axis and y-axis representing the image width and height, respectively, as shown in Figure 3. The size of each bubble indicates the frequency of images with that specific resolution, while the color reflects the distribution density—darker colors signify higher image counts at that resolution.

The results reveal significant diversity in image size within the dataset. The images span a wide range of resolutions from low to high, and their aspect ratios vary considerably. This reflects the lack of standardization in image acquisition, such as differences in device type, shooting distance, and camera angle. This diversity in the data collection process is valuable for training and testing the generalization ability of the LS-FRCNN model.

When considering image width and height together, we observe that the sizes are spread across different groups rather than clustering around specific resolutions. This indicates that the images originate from various sources and were captured under diverse conditions. Additionally, some image sizes are common, while others are relatively rare. This mix of common and rare sizes presents greater challenges for model training but also enhances the model’s performance in complex environments.

**Figure 4:**
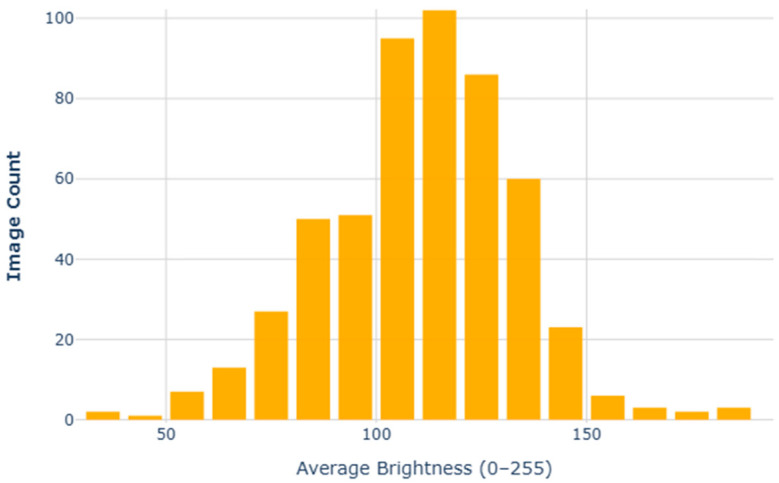
Brightness distribution chart.

In addition, we performed a statistical analysis of image brightness, using the average grayscale value as the brightness estimation metric. Figure 4 presents the histogram distribution of image brightness. The results show that the brightness of images in the dataset is relatively concentrated, primarily within the average brightness range of 100 to 120, indicating that most images have a moderate brightness level. However, the brightness distribution also covers a broad range from dark to bright, reflecting the diversity caused by varying lighting conditions during image capture.

Combined with the manual annotation process, it is clear that some images exhibit significant real-world challenges, such as occlusions, cluttered backgrounds, off-angle views, shadow coverage, or overlapping targets. These uncontrollable factors in real-world scenarios pose challenges for model training while also significantly enhancing the model’s generalization capability and adaptability to complex environments.

To make the model more robust against the interference factors mentioned earlier, this study also used an unsharp masking data augmentation method during training. This helps to highlight the edges and textures in the images, making the model more sensitive to the outlines of plants. More details can be found in Section 2.1.2.

#### 2.1.2. Data Processing

To enhance the edge details and texture features in images, this paper employs the classic sharpening algorithm—the unsharp masking data processing method. This method enhances the high-frequency components in the image, making the image visually clearer and improving the effectiveness of subsequent image processing and feature extraction.

The unsharp masking data processing method is essentially an image sharpening technique based on the concept of Laplacian enhancement. Its steps are as follows:

1. Apply a Gaussian filter to blur the original image I(x,y), obtaining the low-frequency component B(x,y), as shown in Equation (1):

(1) $$B(x,\;y)=G_{\delta }\left(x,\;y)\;*\;I(x,\;y\right)$$

where $G_{\delta }$ denotes a two-dimensional Gaussian kernel with standard deviation δ, and the symbol ∗ represents the convolution operation.

2. Calculate the high-frequency details of the image (i.e., the enhancement term), as shown in Equation (2):

(2) D(x, y)=I(x, y)−B(x, y) 

3. Add the enhancement term back to the original image with a certain intensity k (in the experimental method, k = 1.5), obtaining the sharpened image. The process is shown in Equations (3)–(5):

(3) $$I_{\mathrm{sharp}}(x,\;y)=I(x,\;y)+k\;\cdot \;D(x,\;y)\;$$

(4) $$I_{\mathrm{sharp}}\left(x,\;y)=(1+k)\;\cdot \;I(x,\;y\right)-k\;\cdot \;B(x,\;y)$$

(5) $$I_{\mathrm{sharp}}\left(x,\;y)=2.5\;\cdot \;I(x,\;y\right)-1.5\;\cdot \;B(x,\;y)$$

To put it simply, this method works by finding the difference between the original image and a blurred version of it. This difference, which represents the details of the image, is then added back to the original image in a certain proportion to make the image look sharper.

**Figure 5:**
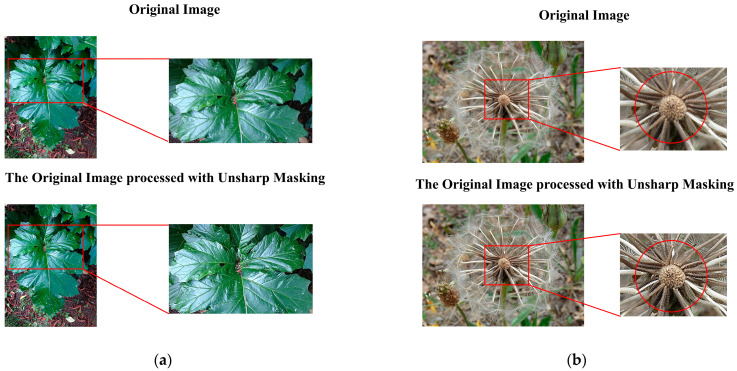
Actual effect of data preprocessing on target texture ((**a**,**b**) represent two typical cases).

To verify the effectiveness of the unsharp masking data processing method in texture enhancement and background differentiation, three groups of images were selected for comparative experiments. The analysis is as follows:

First, regarding texture enhancement effects (Figure 5). In Figure 5a (green plant large leaf), the original image shows smooth leaf texture with blurred edges, especially lacking detail in light-reflective areas. After unsharp masking data processing, the leaf vein structure becomes clearly visible, and the transition between shiny areas and shadows is sharper, giving the overall image a more three-dimensional feel.

In Figure 5b (the dandelion structure), the fiber structure in the center of the original image is rather blurry. After processing, it can be seen that the outline of each filament has been significantly enhanced. At the same time, the part marked by the orange circle in the image can be particularly focused on—the texture in the center of the image is dense but not chaotic, enhancing the layering effect of the biological structure.

The processing results of the aforementioned representative images have verified the effectiveness of the unsharp masking data processing method in enhancing details and clarifying textures, and it is particularly suitable for natural images that require the preservation of complex texture features.

**Figure 6:**
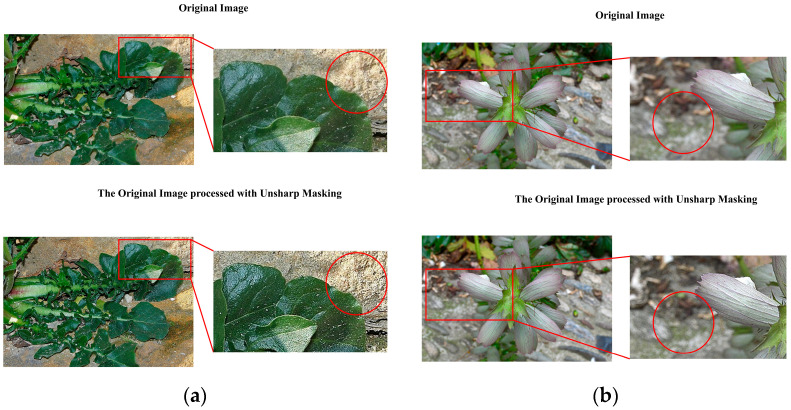
Actual effect of data preprocessing on background ((**a**,**b**) represent two typical cases).

Secondly, regarding the improvement of background differentiation (Figure 6). In Figure 6a (wall and leaf scene), the boundary between the green leaves and the gray-yellow wall background in the original image is not very clear, while the edge transition in the shadow areas is relatively smooth. After unsharp masking data processing, the boundary between the leaf edges and the background becomes clearer, and the granular texture of the wall is enhanced. Pay particular attention to the yellow highlighted area, where the contrast between the target and background is significantly improved after the image processing. In Figure 6b (petals with a background blurred by depth of field), the petal veins in the original image are not very clear, and due to the depth of field effect, the background is relatively blurry and has colors similar to the petals. After unsharp masking data processing, the petal veins become noticeably clearer, and the lighting response is significantly enhanced. Additionally, focusing on the yellow circled area, the background’s granular texture and the contrast between foreground and background are also enhanced.

In order to verify the improvement effect of the data processing method based on the unsharp masking on the Split SAM attention mechanism, we additionally conducted comparative experiments using other sharpening algorithms and color enhancement algorithms to illustrate the results. The dataset used was the self-built dataset used in the ablation experiment, and the model used was LS-FRCNN. The test results are shown in Table 2.

The experimental results indicate that the unsharp masking based data processing method significantly outperforms other enhancement methods. Specifically, although the Laplace operator sharpening can effectively extract edge features, it is sensitive to noise in field environments, leading to interference in edge detection results (mAP 59.13%, mF1 score 0.39). The Sobel operator has advantages in extracting vertical and horizontal boundaries, but due to the complex and variable ecological characteristics of plant structures in natural scenes, its boundary response is unstable, making it difficult to accurately capture foreground targets (mAP 53.21%, mF score1 0.40). The high enhancement filtering (HBF) can enhance the overall image details, but when optimizing the non-sharpening mask, it will excessively amplify background interference, affecting the accuracy of foreground segmentation (mAP 57.14%, mF1 score 0.49). The HSV color space-based enhancement method, which focuses solely on optimizing color features, shows limited improvement in separating the foreground from the background (mAP 57.06%, mF1 score 0.44). This further underscores the unique effectiveness of unsharp masking in enhancing image details rather than colors.

In contrast, the combination of unsharp masking and Split SAM effectively suppresses noise interference while maintaining detail enhancement, achieving the best overall performance (mAP 63.98%, mF1 score 0.47). This validates the unique advantage of the unsharp masking method in activating the SAM attention mechanism—it not only strengthens the representation of target features but also avoids the negative effects introduced by other enhancement methods.

### 2.2. Model Improvements

#### 2.2.1. Lightweight Bottleneck Layer

**Figure 7:**
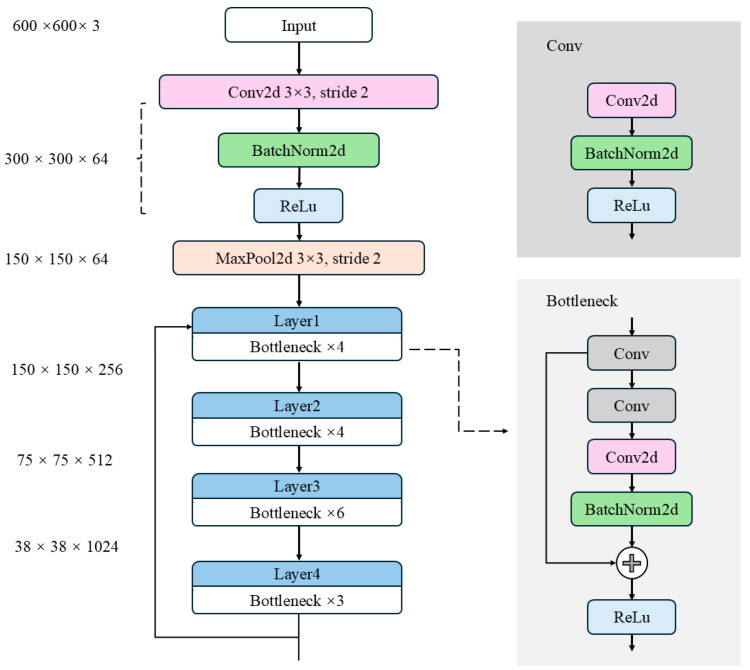
Backbone architecture of the original Faster R-CNN.

As depicted in Figure 7, the original Faster R-CNN backbone network [24] is composed of three key components: the feature extraction network layer, the region proposal network, and the RoI pooling layer. The input image is initially resized to a 600 × 600 × 3 format without loss of information, and then it is passed through the backbone network for feature extraction. In the Faster R-CNN architecture, feature extraction primarily relies on deep neural networks made up of multiple convolutional modules (e.g., Conv Blocks, which include Conv and Identity Blocks as shown in Figure 7, with Conv operating at a stride of 1). These modules are tasked with extracting high-level semantic features from the input image. However, as the model depth and parameter size increase, deploying Faster R-CNN on embedded devices and in real-time applications becomes increasingly challenging.

To tackle this challenge, recent research has focused on developing lightweight network structures. For instance, Zhu et al. [25] demonstrated that depthwise separable convolution significantly reduces the number of parameters and computational costs by applying a single convolution kernel to each channel of the feature map. Additionally, pointwise convolution using 1 × 1 kernels further optimizes computation by integrating channel information. These techniques have worked really well in applications like cashmere and wool fiber recognition. Similarly, many parts of wild plants, like leaves and stems, also have complex textures and loose structures like wool.

Inspired by these findings, this study introduces a lightweight bottleneck structure based on depthwise separable convolution to replace the traditional ResNet bottleneck module. This modification aims to reduce the computational load and model parameters in the Faster R-CNN backbone network. The design of this module incorporates structural optimization strategies commonly used in lightweight networks like MobileNet and ShuffleNet [26,27]. By decoupling convolution calculations across channels and spatial dimensions, this approach significantly reduces resource consumption while maintaining model performance.

**Figure 8:**
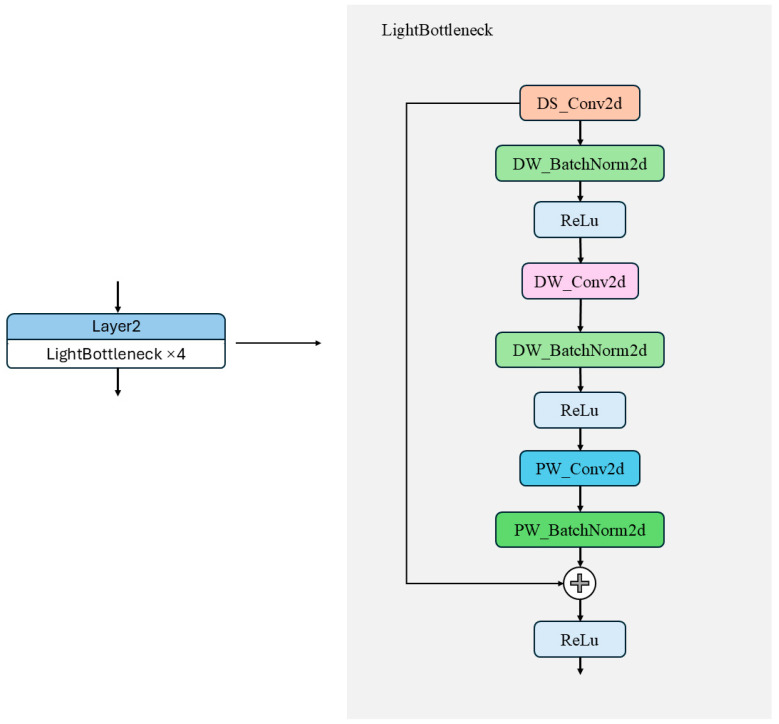
Framework of the light bottleneck module.

Specifically, the lightweight bottleneck module first employs a 1 × 1 depthwise separable convolution combination (DS_Conv2d and DW_BatchNorm2d) to compress the channels of the input feature map, thereby reducing subsequent computation. Next, a 3 × 3 depthwise convolution combination (DW_Conv2d and DW_BatchNorm2d) is applied for spatial feature extraction. This operation performs independent convolution within each channel, significantly reducing computational overhead. Finally, a 1 × 1 pointwise convolution combination (PW_Conv2d and PW_BatchNorm2d) restores the output feature channels to meet the dimensional requirements of the residual connection, as illustrated in Figure 8. Compared to traditional convolution, pointwise convolution has fewer parameters and lower computational cost. By integrating features extracted by multiple convolution kernels at different scales, this structure not only significantly reduces the parameter scale and FLOPs but also maintains strong feature representation capability.

#### 2.2.2. Split SAM Attention Mechanism

Figure 7 illustrates the traditional ResNet architecture, particularly the ResNet50 model that employs bottleneck modules. While residual connections effectively mitigate the vanishing gradient problem and capture multi-level features by progressively increasing the number of channels, several significant issues remain.

First, the fixed receptive field limits the network’s adaptability when handling multi-scale objects, making it unable to flexibly adjust to features of different scales. This restricts the network’s ability to effectively capture both global information and fine-grained details.

Second, the information transmission efficiency is low. Although residual connections promote information flow between layers, there is no effective mechanism to dynamically adjust the attention weights of different levels. This results in insufficient fusion of high-level and low-level features, impairing the network’s ability to express subtle differences.

Finally, the computational complexity is high. While the bottleneck modules effectively improve the performance of deep networks, their gradually increasing computational cost, especially during cross-scale feature fusion, leads to redundant calculations due to the limited receptive field and insufficient information transmission. This impacts the network’s efficiency and inference speed.

These issues pose challenges for ResNet when dealing with complex scenarios and call for improvements in network design.

**Figure 9:**
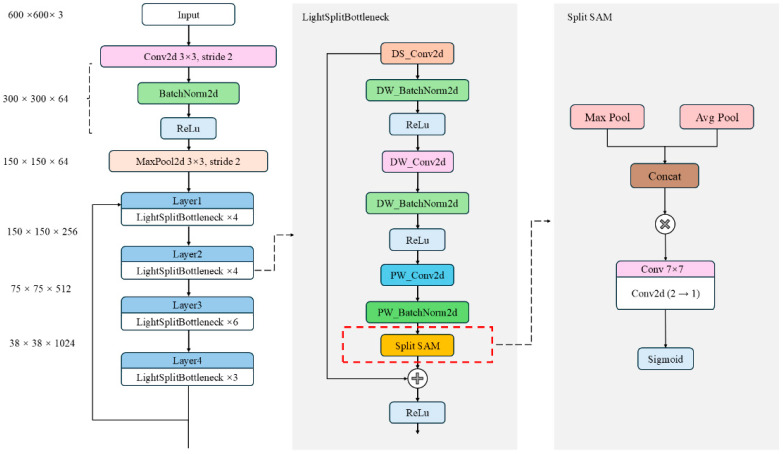
Split SAM improved lightweight feature extraction layer architecture diagram.

To address these problems, we propose an improved network architecture that combines a lightweight bottleneck module with an enhanced spatial attention mechanism module, named Split SAM, as shown in Figure 9. In this architecture, the introduction of Split SAM plays a key role, particularly in dynamically adjusting the focus areas of feature maps and enhancing the separation between foreground and background, highly boosting the network’s performance and efficiency.

By integrating the lightweight bottleneck module with Split SAM, we can enhance the network’s adaptability in multi-scale object detection and complex scenarios while maintaining lightweight computation. This integration also improves the accuracy and efficiency of feature fusion.

The core idea of the Split SAM spatial attention module is to divide the channel dimension of the input feature map into multiple subspaces and independently learn a spatial attention map for each subspace. Let the input feature map be defined as follows:

(6) $$X\in R^{B\times C\times H\times W}$$

Here, B represents the batch size, C is the number of channels, and H and W are the spatial dimensions of the feature map. The channel dimension is divided into G subspaces, with each subspace containing $C_{g}=C/G$ channels:

(7) $$X=\left[X_{1},X_{2},\ldots ,X_{G}\right],X_{g}\in R^{B\times C_{g}\times H\times W}$$

In our design, we set G=1 to reduce computational complexity and memory access cost, which is particularly important for deployment on resource-constrained edge devices. A larger G leads to more convolution operations and fragmented memory access, increasing latency and energy consumption.

For each subspace $X_{g}$, Split SAM extracts the spatial context features of the group using max pooling and average pooling, as follows:

(8) $$F_{g}^{\max}=\mathrm{MaxPool}\left(X_{g}\right),F_{g}^{\mathrm{avg}}=\mathrm{AvgPool}\left(X_{g}\right)$$

The two are concatenated along the channel dimension to obtain a 2D feature map:

(9) $$F_{g}=\left[F_{g}^{\max};F_{g}^{\mathrm{avg}}\right]\in R^{B\times 2\times H\times W}$$

Then, a 7 × 7 convolutional layer is used to extract spatial attention features, and finally a Sigmoid activation function is applied to generate the attention map:

(10) $$M_{g}=\sigma \left(f^{7\times 7}\left(F_{g}\right)\right),M_{g}\in R^{B\times 1\times H\times W}$$

The obtained attention map is then element-wise multiplied with the original subgroup feature map:

(11) X^g=Xg⊙Mg

Finally, the outputs of all subgroups are concatenated back along the channel dimension to obtain the final output.

(12) X^=Concat(X^1,X^2,…,X^G)∈RB×C×H×W

This module only affects the spatial dimensions of local feature maps. It does not change the number of input channels or the spatial resolution. Also, it does not rely on any particular network structure or semantic level. As a lightweight attention module, Split SAM does spatial modeling in each channel group on its own. It does not make the structure more complex and improves the local spatial response. It can be easily added after any convolutional layer in the backbone network. This helps the model better understand spatial details like object edges and textures.

**Figure 10:**
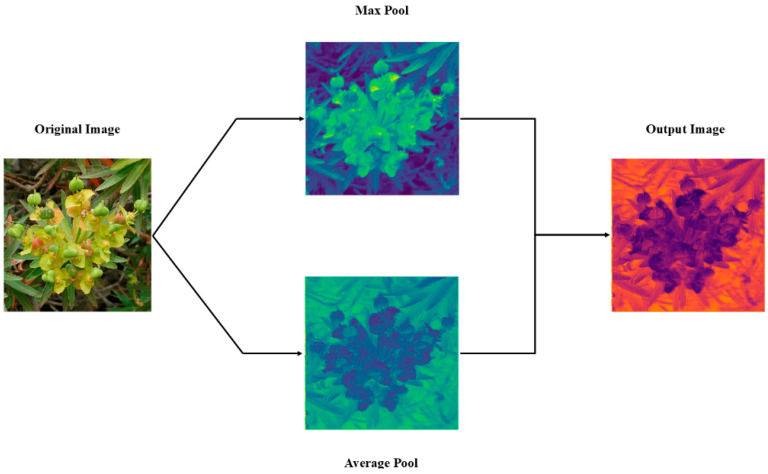
Diagram of feature extraction effects of split spatial attention module (Split SAM).

Figure 10 clearly demonstrates the effect of Split SAM in visualizing the attention map for the original image. From the figure, it can be seen that the original image is first processed through max pooling and average pooling, resulting in two feature maps. The max pooling map highlights the local maximum values in the image, making the foreground more prominent, while the average pooling map shows the smooth features of the image, which will enhance the background features. These two feature maps are then merged to generate an output attention map. The output attention map reflects the importance of different regions in the image, with the highlighted areas indicating the parts where attention is concentrated. In Figure 10, the output attention map has a significant chromatic difference between the foreground (the target plant) and the background (the wild environment), indicating that this processing makes the contrast between the foreground and background in the image more prominent, that is, the main detection and recognition objects are more prominent, thereby enhancing the model’s perception ability for the foreground and background, and improving the detection and recognition accuracy.

**Figure 11:**
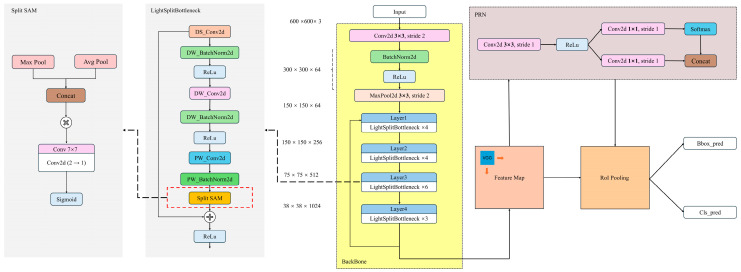
Light Split SAM Faster R-CNN architecture diagram.

After improving the backbone and neck architectures, the framework of the Light Split SAM Faster R-CNN is shown in Figure 11. The main modules and their functions are described as follows:

After improving the backbone feature extraction network, the framework of LS-FRCNN is shown in Figure 11. The main module functions are described as follows:

Proposal Region Proposal Network (RPN): This is a key component of the Faster R-CNN model. It consists of two-dimensional convolutional layers, batch normalization, and activation functions, which are used to identify the regions in the feature map that may contain the target and generate candidate proposals. This approach helps to reduce the number of regions that need to be processed subsequently, thereby improving the detection efficiency.

RoI Pooling Layer: This is a specialized pooling layer that maps candidate regions of varying scales onto fixed-size feature maps, integrating multi-scale feature information to provide a unified input size for subsequent classification and bounding box regression.

Classification and Bounding Box Regression Module: This module consists of two parallel branches. The bbox_pred branch predicts bounding boxes by refining the proposals to accurately localize the objects; the cls_pred branch predicts the object classes using a Softmax function. The final output includes predictions of bounding boxes and class labels.

Compared with the original “Faster R-CNN”, our model differs in the following aspects in terms of improvements:

Firstly, by improving the lightweight bottleneck module, we have addressed the issues of high computational complexity and insufficient sensitivity to fine features in the traditional ResNet50 architecture. The use of depthwise separable convolutions not only reduces the computational load and the number of parameters but also enhances the ability to capture fine features.

Secondly, the introduction of the Split SAM module solves the problems of fixed receptive fields and inadequate foreground–background separation in the original feature extraction architecture. This module can adjust the network’s focus on different areas in a dynamic and efficient way, making the important details stand out more. Because of this, the network gets much better at finding objects of different sizes and recognizing targets against complex backgrounds.

### 2.3. Model Training and Evaluation

To evaluate the performance of ULS-FRCNN in detecting wild plants, we compared it with the original Faster R-CNN model (Origin), the Faster R-CNN model improved with a lightweight bottleneck layer (Baseline), and the lightweight Faster R-CNN model optimized only with the Split SAM attention mechanism (LS-FRCNN). The parameters of the experimental platform used for training and testing are shown in Table 3.

During training, we trained the network using images from the dataset that had been processed with unsharp masking and those that had been preprocessed with sharpened masks. The former, combined with the LS-FRCNN, forms our best-performing architecture, the ULS-FRNN. To ensure the generalizability and reproducibility of our research, we applied a consistent processing method to all subsequent datasets. The overall training parameters of the network are listed in Table 4, and the parameter settings for the Region Proposal Network (RPN) are shown in Table 5.

The performance evaluation metrics include precision (P), recall (R), F1 score, mAP (mean average precision), inference speed, number of parameters (params), and GFLOPs (giga floating-point operations per second).

Among these metrics, P represents the proportion of true positive samples among all predicted positive samples, R reflects the proportion of true positive predictions among all actual positive samples, F1 score is a comprehensive metric used to measure both precision and recall, and mAP represents the mean average precision for object detection at a threshold of 0.5. The formulas for calculating P, R, F1 score, and mAP are shown in Equations (13)–(26), as detailed below:

(13) $$P\;=\;\frac{TP}{TP+FP}$$

(14) $$R=\frac{TP}{TP+FN}$$

(15) $$F1\;\mathrm{score}=\frac{2\times P\times R}{P+R}$$

(16) $$\mathrm{mAP}=\frac{\sum_{c=1}^{C}AP(c)}{C}$$

where *TP*, *FP*, and *FN* represent the numbers of true positives, false positives, and false negatives, respectively; *AP*(*c*) denotes the average precision for a single plant category c; and *C* represents the total number of detection categories. In our self-built dataset, *C* = 5, including *Acanthus mollis* (Bear’s Breeches), *Cirsium eriophorum* (Woolly Thistle), *Gentiana lutea* (Yellow Gentian), *Hyacinthoides non-scripta* (Bluebell), and *Urospermum dalechampii* (False Dandelion).

## 3. Results

### 3.1. Model Training

To achieve a fully lightweight wild plant recognition system, we developed four different training schemes based on Faster R-CNN models. For simplicity in the table, the Unsharp Masking method is abbreviated as “UM”, and this process is referred to as the “UM process.” The details of these training schemes are presented in Table 6.

The Origin scheme represents the original Faster R-CNN model. It has a complex structure and many parameters, so it is hard to use on lightweight devices.

The Baseline scheme introduces lightweight bottleneck layers to modify the Faster R-CNN model, significantly reducing the number of parameters while inevitably compromising accuracy to some degree.

The w/o SAM scheme builds upon the Baseline approach but omits the Split SAM attention mechanism while employing unsharp masking for data optimization.

The w/o UM scheme excludes unsharp masking preprocessing but incorporates the Split SAM attention mechanism. We designate this model as LS-FRCNN, representing the Faster R-CNN model enhanced by Split SAM and the lightweighted bottleneck layers.

The UM & SAM scheme combines both the Split SAM attention mechanism and unsharp masking preprocessing to maximize model performance while maintaining lightweight characteristics. We name this architecture ULS-FRCNN, which means the LS-FRCNN model assisted by unsharp masking data preprocessing. All subsequent discussions will refer to the UM & SAM scheme as the ULS-FRCNN scheme. To thoroughly evaluate these improvement schemes, we will provide detailed analysis of the four approaches in ablation and generalization experiments.

**Figure 12:**
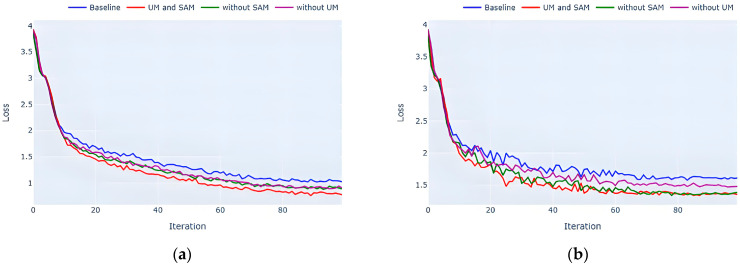
The training loss (**a**) and validation loss (**b**) of four different technical approaches based on the Faster R-CNN model for wild plant recognition.

We conducted comprehensive analyses of training losses across different models and technical approaches on the wild plant dataset. Figure 12a and Figure 12b, respectively illustrate the training loss and validation loss trends for four distinct methods: Baseline (baseline model), UM and SAM (ULS-FRCNN scheme), without SAM (w/o SAM scheme), and without UM (w/o UM scheme, i.e., LS-FRCNN model).

In Figure 12a, the training loss curves of all four methods started high at around 3.9 but diverged as training went on. The ULS-FRCNN scheme quickly reduced loss to about 0.8 after 80 iterations, outperforming the Baseline and single-technique-deficient schemes (w/o SAM or w/o UM). The Baseline model’s loss gradually decreased and stabilized around 1.0. Meanwhile, w/o SAM and w/o UM had similar convergence speeds and fluctuation amplitudes. Notably, in the early training phase (first 10 epochs), these two schemes had lower loss values than the new method but ultimately converged at around 0.9. These results highlight the importance of the Split SAM attention mechanism and unsharp masking preprocessing for optimization efficiency. Overall, the ULS-FRCNN scheme achieved the fastest convergence and maintained the most stable training process, proving the superiority of combining these two techniques.

In Figure 12b, the validation loss trends for wild plant recognition parallel those in Figure 12a. The ULS-FRCNN scheme boasts the lowest loss values and fastest convergence rate. It stabilizes after the 60th epoch, with a final loss of about 1.2. The smooth loss reduction during training indicates excellent generalization capability.

In contrast, the Baseline and w/o UM schemes show overfitting in later training stages (post-60 epochs). Their validation losses paradoxically increase, reaching about 1.6 and 1.5, respectively. This confirms the crucial role of unsharp masking in preventing overfitting. Meanwhile, the w/o SAM approach avoids significant loss rebound but exhibits slower reduction speed and greater fluctuation amplitude, culminating in a final loss around 1.4. This highlights the irreplaceable contribution of Split SAM to model generalization.

Collectively, the ULS-FRCNN scheme achieves the minimal validation loss and the most stable training process, providing conclusive evidence for the effectiveness of this combined strategy.

### 3.2. Ablation Experiments and Comparative Performance Analysis

In computer vision, ablation studies are commonly used to assess the contribution of individual components in a model. By systematically modifying certain modules, we can compare performance differences between the baseline and improved versions. To evaluate the effectiveness of each enhancement in our improved Faster R-CNN model, we conducted ablation experiments using a wild plant dataset containing species such as *Acanthus mollis* (Bear’s Breeches), *Cirsium eriophorum* (Woolly Thistle), *Gentiana lutea* (Yellow Gentian), *Hyacinthoides non-scripta* (Bluebell), and *Urospermum dalechampii* (False Dandelion). The dataset includes both field images and close-up photographs, enabling comprehensive testing across varied visual conditions.

**Figure 13:**
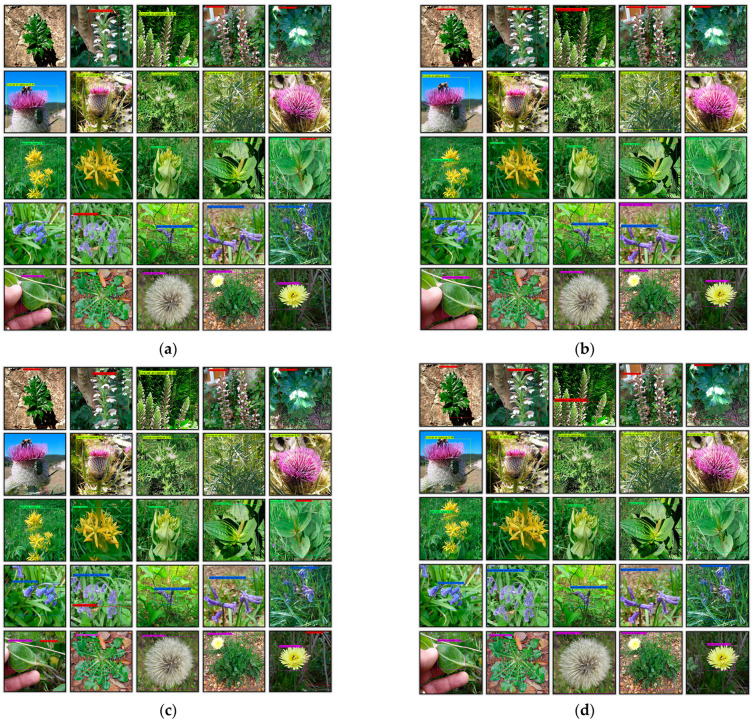
(**a**) Detection results of Baseline scheme, (**b**) detection results of w/o UM scheme, (**c**) detection results of w/o SAM scheme, and (**d**) processing results of ULS-FRCNN scheme.

The quantitative results are summarized in Table 7, and the visual output is presented in Figure 13. Our proposed ULS-FRCNN architecture replaces the original bottleneck convolution layers with depthwise separable convolution modules and integrates the Split SAM spatial attention mechanism into the ResNet50 backbone. This modification brings about a significant performance improvement. Although there is a 5.63% decrease in mean precision (mPrecision) compared to the baseline, the model achieves a 9.07% increase in mean recall (mRecall) and a 12.77% improvement in mean average precision (mAP). It also has the highest mean F1 score (mF1 score) of 0.47 among all tested configurations, which means it has a better balance in detection and fewer missed detections. It should be noted that the above mean precision, mean recall, and mean F1 score are the results of averaging the relevant indicators of each plant in the self-built dataset.

To further verify these gains, we analyzed class-specific precision metrics across five representative plant species, as shown in Table 8, Table 9, Table 10 and Table 11. The ULS-FRCNN scheme demonstrates consistent improvement in average precision across all categories: *Acanthus mollis* (+26.94%), *Cirsium eriophorum* (+4.38%), *Gentiana lutea* (+14.25%), *Hyacinthoides non-scripta* (+6.00%), and *Urospermum dalechampii* (+12.27%). These results support the enhanced feature extraction capability of our improved network.

We also evaluated the effect of using only one of the two enhancements. Adding only the Split SAM module improved model stability and mAP, while unsharp masking alone also increased mAP. However, the test results based solely on a single optimization model showed fluctuations in accuracy, indicating that its performance was not stable. In contrast, the approach that combines UM and SAM methods (ULS-FRCNN scheme) demonstrated the most stable performance across multiple indicators.

Finally, our model significantly reduces computational cost. Compared with the original ResNet50-based Faster R-CNN, the number of parameters decreased by 15.986 million and computation by 616.529 GFLOPs. These reductions make the improved model more suitable for mobile deployment and edge computing applications.

Although our ULS-FRCNN architecture improves recall and mAP, it shows a slight decrease in precision compared to the baseline. This may be due to the baseline overfitting on species like *Cirsium eriophorum*, which has a simple background and bright flowers—resulting in high precision but low recall and F1 score. Our architecture achieves a better balance between these metrics. To reduce false positives in practical applications, adjusting confidence thresholds or applying stricter post-processing could be effective and will be explored in future work.

In summary, the ULS-FRCNN architecture enhances mAP, recall, and stability while also improving model efficiency—making it practical for real-world plant detection tasks.

### 3.3. Comparative Results of Object Detection Models

#### 3.3.1. Comparative Results on Custom Dataset

Figure 13 presents the detection results of four distinct Faster R-CNN-based technical approaches for wild plant recognition. The plant species in these sample images are arranged from top to bottom as follows: *Acanthus mollis* (red bounding boxes), *Cirsium eriophorum* (cyan-yellow boxes), *Gentiana lutea* (green boxes), *Hyacinthoides non-scripta* (blue boxes), and *Urospermum dalechampii* (purple boxes). Images without any colored bounding boxes indicate failed detection cases.

For identical plant sample images, the Baseline Faster R-CNN approach demonstrated the poorest detection performance, with three missed detections and four false positives. After implementing the Split SAM-enhanced architecture, the model showed significant improvement in species identification accuracy, yielding only two errors: one missed detection and one false positive. When using solely unsharp masking processed data, the system produced two missed detections and two false positives. In contrast, the ULS-FRCNN scheme achieved perfect accuracy with zero errors. These results confirm that the integrated Split SAM and Unsharp Masking technique delivers optimal recognition performance in practical detection scenarios.

#### 3.3.2. Generalization Capability Evaluation

To further demonstrate the versatility of ULS-FRCNN architecture, we conducted comparative studies on two different open-source datasets in the agricultural and forestry fields, namely the Edible Wild Plants dataset and the Wild Plants portion of the Wild Edible Plants dataset.

In the experimental design, to ensure the reliability and comparability of results, we adopted uniform model training parameters across all datasets. Simultaneously, we removed data that completely did not conform to the wild plant scenario. Furthermore, to maintain consistency with the training conditions of the self-built dataset and ensure balanced data distribution and proportions, we standardized the data volume for each individual plant species, deleting anomalous and duplicate data to keep it within the range of 100 ± 30 images. Under the condition of meeting the above requirements, the researchers performed random sampling on datasets exceeding five categories to ensure category consistency in the test set. The detailed sample selection is shown in Table 12.

To demonstrate the effectiveness of the optimal solution, this test compared the Baseline scheme and the ULS-FRCNN architecture on two datasets. The detailed comparative results are presented in Table 13.

As can be observed, compared to the baseline model, ULS-FRCNN demonstrates superior performance primarily in mean average precision (mAP), achieving improvements of 7.68% and 11.06% mAP on the aforementioned two datasets, respectively. Furthermore, ULS-FRCNN outperforms the baseline model in terms of mean precision (mPrecision), mean recall (mRecall), and mean F1 score (mF1 score). Overall, these results indicate that compared to the baseline model, the ULS-FRCNN architecture exhibits more excellent performance in wild plant detection and classification tasks.

**Figure 14:**
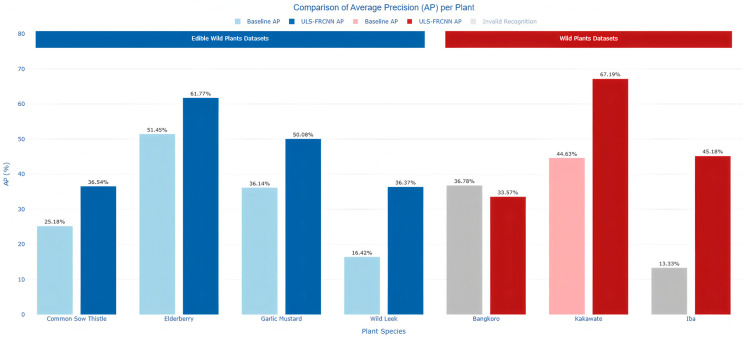
Comparative analysis of average precision (AP) per plant species.

Figure 14 presents the comparison of average precision (AP) between the baseline model (Baseline AP) and the ULS-FRCNN architecture on two datasets: Edible Wild Plants and Wild Plants. The light blue and dark blue bars represent the performance of the baseline model and ULS-FRCNN architecture on the Edible Wild Plants dataset, respectively. The light red and dark red bars correspond to the results of the baseline model and ULS-FRCNN architecture on the Wild Plants dataset. Gray bars indicate AP values for invalid plant recognition. The selected plant species meet the following criteria: at least one model can effectively recognize them, and the ULS-FRCNN architecture achieves an AP improvement exceeding 10%.

On the Edible Wild Plants dataset, the ULS-FRCNN architecture demonstrates significantly better recognition performance than the baseline model for all four plant species. Specifically:

- Common Sow Thistle’s AP increases from 25.18% to 36.54%, an absolute improvement of 11.36%;
- Elderberry’s AP increases from 51.45% to 61.77%, an absolute improvement of 10.32%;
- Garlic Mustard’s AP rises from 36.14% to 50.08%, an absolute improvement of 13.94%;
- Wild Leek shows the most remarkable accuracy improvement, with AP surging from 16.42% to 36.37%, representing a relative increase of 121.5%.

On the Wild Plants dataset, the ULS-FRCNN architecture exhibits even more groundbreaking improvements:

- Bangkoro achieves a qualitative leap from complete recognition failure to effective recognition with AP = 33.57%;
- Iba similarly accomplishes a transformative improvement from invalid recognition to high-precision recognition with AP = 45.18%;
- Kakawate’s AP increases from 44.63% to 67.19%, an absolute improvement of 22.56%.

These results highlight three core breakthroughs of the ULS-FRCNN architecture:

1. Achieving recognition breakthroughs from invalid to valid: For species like Bangkoro and Iba that the baseline model completely failed to recognize, ULS-FRCNN architecture achieves effective detection for the first time (AP > 30%), filling the recognition blind spots of the original model;
2. Ultra-high growth rate from low baselines: Species like Wild Leek with low baseline AP achieve relative improvements exceeding 100%, proving the model’s exceptional capability in optimizing hard-to-recognize samples;
3. Stable cross-scenario improvements: Maintaining >10% absolute improvements on both edible plants and general wild plant datasets, verifying the algorithm’s generalization robustness.

The ULS-FRCNN architecture significantly boosts recognition accuracy and greatly widens the range of recognizable species. This makes it highly suitable for comprehensive recognition tasks like biodiversity monitoring and wilderness survival assistance. Meanwhile, it uses lightweight data processing techniques such as unsharp masking, and designs lightweight bottleneck layers and Split SAM. These ensure that ULS-FRCNN maintains similar parameter scale, training cost, and inference speed to the baseline model. It achieves efficient recognition of more species while keeping high efficiency, thus enhancing the model’s practicality and cost-effectiveness.

### 3.4. Simulated Performance Evaluation of Object Detection Models on Edge Devices

To simulate the performance of LS-FRCNN and Unsharp Masking on a representative edge device, we selected the NVIDIA Jetson Nano Developer Kit as the target hardware. According to NVIDIA’s official specifications, the Jetson Nano is equipped with a quad-core ARM Cortex-A57 CPU (1.43 GHz), a 128-core Maxwell GPU (472 GFLOPS), and 4 GB LPDDR4 memory, with a typical power envelope of 5–10 W. This configuration represents a typical low-power embedded AI device widely used in real-time edge computing applications.

To closely mimic the hardware limitations of Jetson Nano and ensure consistency with the YOLOv5n model configuration, the LS-FRCNN model and Unsharp Masking algorithm were tested under the following environment and settings:

- Local device: Intel Core i7-11800H (2.3 GHz, 8 cores, 16 threads);
- Simulation setup: GPU acceleration disabled, maximum number of threads limited to 4: this was done to better approximate the computational constraints of the Jetson Nano device, which has a lower-end GPU and limited CPU cores;
- Framework: We used PyTorch because both our LS-FRCNN and the YOLOv5n baseline are based on it, ensuring fair comparison;
- Input data: The input images are resized to 600 × 600 RGB to keep the test conditions consistent with YOLOv5n;
- Testing method: Randomly selected at least 25 images for inference, recorded the time for each image, calculated the average inference time $T_{base}$, and derived the base frame rate $FPS_{base}$. The final result showed a local inference frame rate of 0.08 FPS, corresponding to an average time of 12.5 s per image.

To simulate inference time on the edge device from local CPU measurements, we introduced a performance scaling factor defined as the ratio of the target device frame rate ${\mathrm{FPS}}_{\mathrm{device}}$ to the local test frame rate ${\mathrm{FPS}}_{\mathrm{base}}$:

(17) $$\mathrm{Speed}\text{-}\mathrm{up}=\frac{{\mathrm{FPS}}_{\mathrm{device}}}{{\mathrm{FPS}}_{\mathrm{base}}}$$

Based on published reports and community test data, the Faster R-CNN model without any acceleration optimization (such as TensorRT or quantization) runs at about 0.5 FPS on the Jetson Nano (GPU mode). Using this value, the basic performance scaling factor can be calculated as follows:

(18) $${\mathrm{Speed}\text{-}\mathrm{up}}_{1}=\frac{{\mathrm{FPS}}_{Jetson\;Nano}}{{\mathrm{FPS}}_{base}}=\frac{0.5}{0.08}=6.25$$

This factor also represents the performance scaling for the Unsharp Masking algorithm on Jetson Nano.

Since the proposed LS-FRCNN model reduces parameters by about 57% and computational complexity (GFLOPs) by about 65% compared to the original Faster R-CNN while maintaining accuracy, a structural complexity scaling factor is introduced:

(19) $$r=0.35\Rightarrow {\mathrm{Speed}\text{-}\mathrm{up}}_{2}=\frac{{\mathrm{Speed}\text{-}\mathrm{up}}_{1}}{r}=\frac{6.25}{0.35}\approx 17.86$$

Using the above scaling factor and the local device’s measured frame rate, the estimated inference frame rate of the LS-FRCNN model on the Jetson Nano is as follows:

(20) $${FPS}_{simulated}={FPS}_{base}\times {Speed\text{-}up}_{2}=0.08\times 17.86\approx 1.43\;FPS$$

The actual measured result is approximately 1.41 FPS, which is highly consistent with the simulation, indicating that this inference simulation testing scheme can effectively predict the running performance of LS-FRCNN on Jetson Nano without any TensorRT or quantization acceleration. This frame rate is significantly better than the original Faster R-CNN on the same device.

Furthermore, when compared with the mainstream lightweight detector YOLOv5n (Jetson Nano GPU mode, frame rate about 12.34 FPS, inference latency under 100 ms), LS-FRCNN has a relatively lower inference frame rate but shows significant advantages in detection accuracy (mAP). Although real-time capability is slightly sacrificed, the accuracy improves by about 10.35%, making it suitable for edge applications that require higher detection quality.

Additionally, as an image enhancement method, the Unsharp Masking algorithm demonstrates extremely high inference speed (about 4588 FPS) with an inference latency of only 0.22 ms and very low memory usage (about 44.5 MB). This algorithm effectively enhances image edges and details, which helps improve the accuracy of subsequent object detection models with very low computational cost, making it well-suited for embedded and real-time processing scenarios.

The final performance comparison is shown in Table 14.

## 4. Discussion

Many previous studies have been dedicated to performing data augmentation operations, lightweighting, or improving detection speed to optimize the performance of the target detection algorithm [28,29,30]. However, the performance indicators of the previous studies are unbalanced in terms of model complexity and model recognition accuracy, and they ignore the issue of data processing difficulty. For forestry equipment with strict requirements and low cost, it is crucial to optimize multiple indicators and use efficient, simple data processing methods to cut training costs and boost forestry task efficiency. Considering these strict requirements, we propose a wild plant detection and classification algorithm named ULS-FRCNN, aiming to achieve a balance among model complexity, model recognition accuracy, and data processing difficulty. ULS-FRCNN is derived from Faster R-CNN, featuring a lightweight optimized bottleneck layer and an improved foreground-background segmentation attention mechanism called Split SAM for feature fusion network, along with a simple and efficient unsharp masking data preprocessing method. The results show that ULS-FRCNN achieves 63.98% mAP on five wild plants with only 12 M parameters, performing excellently and promising deployment on low-power platforms.

Firstly, the improvement in computational efficiency performance is mainly due to the adoption of a lightweight bottleneck layer design. As shown in Table 7, the modification of the backbone results in a significant reduction in parameters and GFLOPs. This optimization effect mainly stems from the structural design of the lightweight bottleneck module, as shown in Figure 8. This module effectively improves the running efficiency of the model by reducing redundant computations and optimizing the feature extraction process. Specifically, the lightweight bottleneck module first compresses the input feature map using a 1 × 1 pointwise convolution, reducing the subsequent computational load. It then applies a 3 × 3 depthwise convolution for spatial feature extraction, operating independently in each channel to significantly cut computational costs. Lastly, it uses a 1 × 1 pointwise convolution to restore the output feature’s channel number to meet the residual connection’s dimension requirements. Compared to traditional convolution, this design has fewer parameters and lower computational costs, and it fuses features from multiple convolution kernels at different scales to achieve better image representation.

Secondly, the improvement is partly attributed to Split SAM. The feature extraction ability of the model can be significantly enhanced through the optimized design of Split SAM. As shown in Figure 9, Split SAM first performs global max pooling and global average pooling operations on the input feature map, extracting the maximum response and average response of the feature map, respectively. Then, these two responses are concatenated along the channel dimension and a 1 × 1 convolutional layer generates an attention map. This attention map is normalized using the Sigmoid function and finally multiplied element-wise with the input feature map to achieve weighted operations on the feature map. This approach not only highlights important features but also suppresses irrelevant features, thereby improving the model’s ability to perceive key information. The role of Split SAM lies in enabling the model to dynamically allocate computing resources, focusing on the key regions of the foreground and background in the image. This mechanism is similar to the attention mechanism in the human visual system, effectively improving the model’s ability to parse complex images. Meanwhile, Split SAM effectively improves the model’s running efficiency by reducing redundant computations and optimizing the feature extraction process.

Although ULS-FRCNN has certain advantages in the detection and recognition of wild plants, it still faces many challenges in practical applications. Firstly, the morphological features of wild plants show significant diversity in different growth environments and stages, lacking a unified biological form commonality. Secondly, when agricultural or forestry equipment collects images, it is difficult to obtain high-quality image data from multiple angles and fixed features (such as leaves, flowers, etc.). Moreover, plants may exhibit completely different biological characteristics due to wilting, diseases, or pests during growth, further increasing the difficulty of detection and recognition. In extreme cases, such as when the target is severely occluded, the color is highly similar to the background, or key features are missing, ULS-FRCNN is prone to missing detections or incorrect identifications.

Another limitation stems from the dataset itself. The current core dataset only includes five wild plant species, which restricts its diversity and may hinder the generalization ability of the model to unseen species. Furthermore, the overall dataset size remains relatively small, increasing the risk of overfitting and reducing the model’s robustness under real-world environmental variation. In addition, the collection of wild plant data is inherently challenging due to the need for capturing multiple views and distinctive features (such as leaves, flowers, and stems), all while dealing with environmental variables like lighting, occlusion, and biological changes caused by aging or disease.

To address these issues, future research will focus on several key directions to enhance dataset quality and algorithm robustness. First, we plan to increase the number of wild plant species in the dataset, thereby improving its biological diversity and enabling the model to learn more varied morphological characteristics. Second, we will collect images from multiple perspectives and ensure coverage of critical plant parts, allowing the model to develop a more comprehensive understanding of plant structure. Finally, advanced data augmentation techniques—including random cropping, rotation, flipping, and color jittering—will be employed to artificially diversify the dataset, improve model robustness to appearance variations, and reduce overfitting. In addition, considering practical scenarios where false positives are critical, such as biodiversity conservation, we plan to explore adjusting confidence thresholds and applying stricter post-processing methods to reduce false alarms and enhance model usability. These improvements are expected to significantly enhance the practical applicability and scalability of ULS-FRCNN in complex and resource-limited forestry environments.

## 5. Conclusions

This study proposes a systematic lightweight solution for plant recognition in complex wild environments. It includes comprehensive optimizations from data pre-processing to model architecture, which significantly enhance both efficiency and accuracy. Specifically, we design and implement four technical innovations based on the Faster R-CNN framework, including sharpened mask-based data enhancement, lightweight bottleneck redesign, and Split SAM attention integration. Employing a two-stage detection-recognition strategy tailored for fine-grained botanical structures, our method achieves precise plant identification in challenging field scenarios. The ULS-FRCNN approach—integrating unsharp masking data augmentation, lightweight bottleneck layers, and Split SAM attention mechanism—demonstrates optimal performance, attaining 63.98% mAP in plant recognition tasks while maintaining superior average precision (with the highest average recall and an F1 score of 0.48), confirming the model’s exceptional stability and robustness.

Faster R-CNN is a classic two-stage detection framework that achieves end-to-end learning for region proposal generation and target detection through the Region Proposal Network (RPN), thereby achieving high-precision detection results. The Split SAM enhancement strategy we proposed effectively enhances the model’s perception ability in complex backgrounds. When combined with unsharp masking preprocessing, this method not only enhances the representation of image features but also fully exploits the potential of Split SAM—being able to accurately capture the subtle features of plants in cluttered backgrounds—while maintaining the lightweight characteristics of the entire process. This solution demonstrates excellent performance in lightweight design, significantly reducing computational requirements without sacrificing recognition performance, thus making it suitable for deployment in real-time and resource-constrained conditions such as edge computing and field surveys.

The improvements explored in this study not only substantially advance plant recognition accuracy and efficiency but also provide critical technical references for deploying edge computing in agricultural/forestry applications. By combining lightweight deep learning architecture with biologically informed preprocessing techniques, successful implementation of this method will accelerate the adoption of plant recognition technology in ecological monitoring, biodiversity research, and species conservation, establishing essential technical support for interdisciplinary research advancements.

## Figures and Tables

**Table 1:** Composition of the field plant dataset.

Datasets	Variety	Number	Total
Training set	*Acanthus mollis*	87	429
	*Cirsium eriophorum*	77	
	*Gentiana lutea*	85	
	*Hyacinthoides non-scripta*	78	
	*Urospermum dalechampii*	102	
Validation set	*Acanthus mollis*	8	48
	*Cirsium eriophorum*	9	
	*Gentiana lutea*	9	
	*Hyacinthoides non-scripta*	13	
	*Urospermum dalechampii*	9	
Test set	*Acanthus mollis*	15	54
	*Cirsium eriophorum*	9	
	*Gentiana lutea*	9	
	*Hyacinthoides non-scripta*	8	
	*Urospermum dalechampii*	13	

**Table 2:** Comparison of test results based on different data augmentation methods.

Methods	mAP	mPrecision	mRecall	mF1 Score
Unsharp Masking	63.98%	39.27%	65.75%	0.47
Laplace	59.13%	29.46%	59.59%	0.39
Sobel	53.21%	35.14%	55.50%	0.40
HBF	57.14%	35.30%	64.21%	0.49
HSV-V	57.06%	35.04%	63.98%	0.44

**Table 3:** Experimental platform environment used for training based on the Faster R-CNN model.

Hardware	Parameter
CPU	Intel(R) Core(TM) i7-11800H @ 2.30 GHz
GPU	NVIDIA GeForce RTX 3070 8 GB
RAM	16 G
Operating System	Windows 10
Deep learning framework	Pytorch 1.7.1
Cuda Version	Cuda 11.0
Programming Language	Python 3.7
Integrated development environment	VScode
Package management tools	Anaconda

**Table 4:** Parameter settings for the experimental platform environment used for training based on the Faster R-CNN model.

Hyperparameter	Value
Input image size	600 × 600
Initial learning rate	0.0001
Batch size	4
Epoch	100
Optimizer	ADAM
Momentum	0.9
Weigh decay	0.0005

**Table 5:** Parameter and hyperparameter settings in RPN model training.

Hyperparameter	Value
Ratios	[0.5, 1, 2]
Anchor scales	[8, 16, 32]
Feat stride	16
RPN NMS IoU Threshold	0.7

**Table 6:** Training scheme configurations.

Scheme	Methods		
	Light BTNK	UM Process	Split SAM
Origin			
Baseline	✓		
w/o SAM	✓	✓	
w/o UM	✓		✓
UM & SAM	✓	✓	✓

**Table 7:** Ablation experiments results.

Scheme	Methods			Results					
	Light BTNK	UM Process	Split SAM	mAP	Params	GFLOPs	mPrecision	mRecall	mF1 Score
Origin				-	28.316 M	940.972	-	-	-
Baseline	✓			51.21%	12.392 M	324.406	44.90%	56.68%	0.46
w/o SAM	✓	✓		56.88%	12.329 M		38.69%	60.68%	0.43
w/o UM	✓		✓	56.64%	12.330 M	324.443	39.30%	61.99%	0.45
ULS-FRCNN	✓	✓	✓	63.98%			39.27%	65.75%	0.47

**Table 8:** Single-class plant recognition accuracy results using baseline model processing.

Scheme	All Kinds of Indicators				
	Plant Species	AP	Precision	Recall	F1 Score
Baseline	*Acanthus mollis*	51.66%	32.00%	80.00%	0.46
	*Cirsium eriophorum*	63.95%	80.00%	44.44%	0.57
	*Gentiana lutea*	39.05%	33.33%	53.85%	0.41
	*Hyacinthoides non-scripta*	40.91%	41.67%	38.46%	0.40
	*Urospermum dalechampii*	60.47%	37.50%	66.67%	0.48

**Table 9:** Single-class plant recognition accuracy results using only unsharp masking processing.

Scheme	All Kinds of Indicators				
	Plant Species	AP	Precision	Recall	F1 Score
w/o SAM	*Acanthus mollis*	67.49%	33.33%	100.00%	0.50
	*Cirsium eriophorum*	53.60%	66.67%	44.44%	0.53
	*Gentiana lutea*	47.27%	25.00%	53.85%	0.34
	*Hyacinthoides non-scripta*	42.85%	38.46%	38.46%	0.38
	*Urospermum dalechampii*	73.19%	30.00%	66.67%	0.41

**Table 10:** Single-class plant recognition accuracy results using only Split SAM processing.

Scheme	All Kinds of Indicators				
	Plant Species	AP	Precision	Recall	F1 Score
w/o UM	*Acanthus mollis*	71.93%	38.10%	80.00%	0.52
	*Cirsium eriophorum*	63.62%	83.33%	55.56%	0.67
	*Gentiana lutea*	42.96%	21.88%	53.85%	0.31
	*Hyacinthoides non-scripta*	33.21%	25.93%	53.85%	0.35
	*Urospermum dalechampii*	71.64%	27.27%	66.67%	0.39

**Table 11:** Single-class plant recognition accuracy results using ULS-FRCNN processing.

Scheme	All Kinds of Indicators				
	Plant Species	AP	Precision	Recall	F1 Score
ULS-FRCNN	*Acanthus mollis*	78.60%	40.00%	80.00%	0.53
	*Cirsium eriophorum*	68.33%	75.00%	66.67%	0.71
	*Gentiana lutea*	53.30%	20.59%	53.85%	0.30
	*Hyacinthoides non-scripta*	46.91%	30.77%	61.54%	0.41
	*Urospermum dalechampii*	72.74%	30.00%	66.67%	0.41

**Table 12:** Final data selection across different datasets.

Datasets	Plant Category
Edible Wild Plants	Elderberry
	Garlic Mustard
	Wild Leek
	Common Sow Thistle
	Toothwort
Wild Plants	Bangkoro
	Bignai
	Iba
	Kakawate
	Sunflower

**Table 13:** Comparison of test results across different datasets.

Datasets	Methods	mAP	mPrecision	mRecall	mF1 Score
Edible Wild Plants	Baseline	33.11%	35.38%	21.50%	0.254
	ULS-FRCNN	40.79%	38.56%	41.79%	0.396
Wild Plants	Baseline	42.29%	20.00%	21.67%	0.162
	ULS-RCNN	53.35%	56.86%	42.04%	0.44

**Table 14:** Inference performance comparison of LS-FRCNN, Unsharp Masking, and YOLOv5n on Jetson Nano.

Model/Method	Performance Metrics				
	mAP	Params	FPS	Memory Usage	Inference Time
Yolov5n	46.30%	1.775 M	12.34	≤2000 MB	≤100 ms
LS-FRCNN	56.64%	12.330 M	≈1.41	2624.02 MB	710.32 ms
Unsharp Masking	-	-	4588.63	44.49 MB	0.22 ms

## Data Availability

The data utilized in this study were sourced from the LifeCLEF 2015 Plant task database (available at: https://www.imageclef.org/lifeclef/2015/plant (accessed on 20 July 2025)). Our analysis focused on a subset of this database, selected based on the criteria of the five plant species described in the manuscript. The subset data were processed according to the methods outlined in our study. The processed datasets are available from the first author upon reasonable request. For access to the specific data used in this research, please contact zxtsui@bjfu.edu.cn.
